# Self-determination theory and the influence of social support, self-regulated learning, and flow experience on student learning engagement in self-directed e-learning

**DOI:** 10.3389/fpsyg.2025.1545980

**Published:** 2025-03-26

**Authors:** Yanhua Yang, Jianfeng Chen, Xin Zhuang

**Affiliations:** ^1^School of Data Engineering, Tianjin University of Finance and Economics Pearl River College, Tianjin, China; ^2^School of Mechanical Engineering, Shenyang University of Technology, Shenyang, Liaoning, China; ^3^Information Engineering Department, Heilongjiang Jiaotong Polytechnic, Haerbin, Heilongjiang, China

**Keywords:** flow experience, learning engagement, self-determination theory, self-regulated learning, social support

## Abstract

E-learning significantly broadens the scope for students to participate in extracurricular self-directed learning to achieve personal goals. However, the existing research has somewhat overlooked this particular setting. Our study delves into how self-determination theory shapes student engagement in learning, influenced by social support, flow experience, and self-regulated learning, while also examining the mediating role of self-determination theory. We gathered 593 questionnaires from students across various disciplines and levels of learning in five Chinese universities. Through structural equation model, we tested 13 hypotheses and several mediating effects basing on self-determination theory. Our findings revealed that social support can predict relatedness, while both flow experience and self-regulated learning have significant impacts on the three basic psychological needs. Furthermore, we observed that competence and relatedness have impacts on the motivation of self-determination theory. A significant positive association exists between motivation and student learning engagement, and insignificant direct pathways have no indirect effect on mediating effects. We discussed the similarities and differences between out-of-class self-directed e-learning and traditional in-class e-learning, considering the same influencing factors. We also offered constructive insights for students to effectively reaching their personal goals.

## Introduction

In today's educational landscape, students increasingly benefit from diverse learning opportunities, yet they also face challenges posed by information and communication technology (ICT), including cognitive disabilities, environmental disconnection, and behavioral inefficiencies. Electronic learning (e-learning)—encompassing online learning, distance learning, and massive open online courses (MOOCs) (Çebi, 2023; He et al., 2023; Qiu et al., 2022)—provides viable solutions to these obstacles. While scholars may lack a universally agreed-upon definition, e-learning generally refers to the fusion of Internet-based digital learning resources and techniques via electronic devices, making it an effective learning approach (Gama et al., 2022). E-learning enhances educational outcomes by offering temporal and spatial flexibility compared to traditional face-to-face educational settings (El-Sabagh, 2021; Sørebø et al., 2009). In China, network learning spaces constitute a pivotal element of the government's *Action Plan for Education Informatisation 2.0*, with over 72 million spaces created and 10 million registered in the real-name identity system (Guo and Zheng, 2019). The COVID-19 pandemic prompted a significant shift toward online learning, with more than 90% of Chinese underage students transitioning to online learning due to lockdowns (Gao et al., 2023). Even post-pandemic, e-learning continued to expand, prompting research endeavors aimed at enhancing student outcomes. For instance, Wang et al. (2021) found that students with high self-efficacy in e-learning can adopt tailored learning strategies to improve their performance. Shi et al. (2020) indicated that fragmented content can be effectively and systematically learned through e-learning using a knowledge graph-based approach. ICT self-efficacy, motivation, and goal-setting—both long-term and short-term—play pivotal roles in optimizing e-learning experiences (Al-araibi et al., 2018; Bai et al., 2022). Çebi (2023) reported that concrete motivations are an essential predictor of e-learning engagement. Qiu et al. (2022) innovatively categorized e-learning behaviors based on distinct rules, uncovering potential connections between e-learning behaviors across different categories. Firat et al. (2017) found that students engaged in e-learning if their intrinsic motivation and self-initiative were maintained. He et al. (2023) noted that the significant impact of educational support on e-learning acceptance, while also emphasizing the importance of emotional support in the process.

Research indicates that students' learning engagement is shaped by multiple factors (Chai et al., 2023; Chiu, 2021; El-Sabagh, 2021; Fredricks et al., 2004; Gao et al., 2023; Jung and Lee, 2018; Lai et al., 2021; Lam et al., 2016; Liao et al., 2023; Moon and Ke, 2019; Shernoff et al., 2003; Sun and Rueda, 2012). However, these studies mostly focused on traditional school settings, with limited attention given to e-learning scenarios where students establish their own learning goals. Our study makes a distinctive contribution by segmenting e-learning into two categories: *in-class e-learning* (ICEL) and *out-of-class e-learning* (OCEL). ICEL refers to classroom-based e-learning, whereas OCEL involves students independently pursuing personal goals, such as exam preparation, competitions, or skill enhancement outside the classroom. Using *self-determination theory* (SDT) as the conceptual framework, our study investigates the influence of *social support, flow experience*, and *self-regulated learning* (SRL) on student engagement in OCEL. Furthermore, this study also contrasts previous research on ICEL with our current OCEL research, identifying similarities and differences. By doing so, we offer strategies for students to enhance learning engagement, and further achieve personal goals.

Specifically, we emphasize that according to SDT, students have three basic psychological needs: competence, relatedness, and autonomy. These needs are crucial for stimulating students' learning motivation and engagement. In the context of OCEL, where students need to set their own goals and manage the learning process autonomously, meeting these basic needs becomes particularly important. This study explores how *social support, flow experience*, and SRL influence these three basic psychological needs of students in the OCEL environment, and further analyze how these needs affect students' learning motivation and engagement.

In ICEL, students typically navigate e-learning with the guidance of teachers; conversely, OCEL, driven by students themselves, may be respond differently to the same influencing factors compared to ICEL. Our study considered OCEL behavior initiated by students themselves rather than passive acceptance of ICEL, which requires students to have higher self-regulated competence. Given the inherent flexibility of OCEL, students experience greater autonomy, prompting our focus on the factors that shape OCEL engagement. Although previous studies have extensively explored e-learning environments, research on self-directed learning in OCEL remains relatively limited.

We identified the antecedents that impact students' self-directed learning and cultivating motivation to promote their engagement in OCEL, and addressed the following issues:

- We explored the influence of social support, flow experience, and SRL on students' three basic psychological needs according to SDT—*competence, relatedness*, and *autonomy*—in OCEL, along with the associated adaptive strategies.
- We explored the differences in how these psychological needs affect learning motivation in OCEL and ICEL, along with proposed solutions.

- We investigated the impact of motivation on student learning engagement in OCEL while combining the influencing relationships, and proposed strategies to enhance it.
- We explored the mediating role of social support, flow experience, and SRL in student learning engagement in OCEL through the three psychological needs of SDT, with a comparison of its direct effects.

The rest of the article is structured as follows. First, the literature review examines SDT and its relationship with social support, flow experience, and SRL. Then, we detail the impact relationship between social support, flow experience, SRL, SDT, and learning engagement, leading into the research hypotheses. Following a comprehensive overview of the research methods, we discuss the findings, and present the research contributions and implications.

### Theoretical framework

SDT emphasizes the development of self-determined abilities (Osei and Bjorklund, 2024), making it a suitable framework for OCEL. In OCEL, students independently plan learning tasks, content, and strategies, assuming ownership of their learning goals. This thoughtful process transitions *amotivation* to *extrinsic*, ultimately culminating in *intrinsic motivation*. When students accept responsibility for their goals, their motivation becomes intrinsic, aligning with SDT.

#### Self-determination theory

Roca and Gagné (2008) were pioneers in applying SDT to e-learning research. According to this theory, students naturally pursue growth and improvement, but achieving this depends on internalizing external motivation by fulfilling three basic psychological needs—*competence, relatedness*, and *autonomy* (Deci and Ryan, 2013). Not all learning activities can be internalized to form motivation; internalization requires individuals to challenge themselves (Vasconcellos et al., 2020). SDT proposes that all three needs exist, and the relationships among *competence, relatedness*, and *autonomy* are equal (Sahin and Yildiz, 2024). Each need is essential, and any deficiency can hinder internalization (Van den Broeck et al., 2016). In this study, relatedness refers to students' sense of support from others; competence refers to their ability to master OCEL and assimilating new knowledge; and autonomy refers to self-regulation. The fulfillment of these psychological needs strengthens motivation, while unmet needs prompt self-regulation based on controllable factors (Vasconcellos et al., 2020).

#### Self-regulated learning

SDT stresses the three basic psychological needs—*competence, relatedness*, and *autonomy*—without pointing out how or what individuals can fulfill them. Van den Broeck et al. (2016) identified various antecedents and outcomes linked to these needs. In OCEL, SRL should be considered one of them, providing strategies for self-control, self-motivation beliefs, and self-judgement (Zimmerman, 1990), transforming mental needs into learning behavior through self-directed processes (Zimmerman, 2008). Scholars have established several SRL models, among which three were developed by Zimmerman (1989), Zimmerman and Campillo (2003), and Zimmerman and Moyla (2009) and are widely applied across e-learning scenarios. Zimmerman was one of the pioneers of SRL (Panadero, 2017). Originating from a social–cognitive perspective, the model is divided into the following three phases: (1) forethought phase, where students set goals and devise strategies; (2) performance phase, where students regulate their bodies and minds and adjust their self-awareness and behavior accordingly; and (3) self-reflection phase, where students engage in self-judgement and self-reaction and identify influencing factors to establish a self-adaptive state (Wong et al., 2018), evaluate their short-term tasks, and summarize their successes or failures (Panadero, 2017). These phases form a cyclical process during the learning process, repeatedly reproducing each other. SRL skills can empower students to become self-directed learners, yielding lifelong benefits (Gabriel et al., 2020). It encompasses cognitive, metacognitive, behavioral, motivational, and emotional aspects of learning (Panadero, 2017) that are related to students' basic psychological needs (Vasconcellos et al., 2020).

#### Flow experience

Flow is a mental state, wherein an individual is fully immersed in an activity, finding it inherently rewarding irrespective of the end result (Czikszentmihalyi, 1990). The following nine conditions encompass the flow experience:

- *Clear goals*: Task goals are unambiguous, and individuals are aware of the specific results they want to achieve.
- *Immediate feedback*: Activities provide immediate feedback, enabling individuals to closely monitor their progress toward their goals.
- *Skill–challenge balance*: Opportunities for action are balanced with individuals' abilities, tasks and activities can be realistically completed.
- *Deep concentration*: Individuals focus with undivided attention on the task at hand.
- *Serenity*: Individuals are single-minded, free of distractions unrelated to the activity.
- *Personal control*: Individuals effectively self-regulate the activity.
- *Reduced self-consciousness*: Individuals are fully engaged in the activity, not their own interests.
- *Altered sense of time*: Individuals' subjective awareness of time duration is transformed.
- *Autotelic experience*: Individuals are intrinsically motivated to engage in the activity, regardless of the outcome.

If these nine conditions are satisfied, individuals experience flow. Flow is a strikingly similar experience to that in which students are engaged in e-learning for a specific task, fully immersed in the activity, concentrating on what they are doing, and gain pleasure not from potential rewards but from the learning experience itself (Esteban-Millat et al., 2018). In OCEL, students engage in self-directed learning to complete certain tasks, exerting their full capacities, with the experience itself serving as their reward (Shernoff et al., 2003). Scholars have posited that when individuals engage in desired tasks that match their abilities, they experience flow **(**Csikszentmihalyi, 1997; Shernoff et al., 2003). Perceiving a balance between task challenges and their own skills empowers students to control their learning and increase their level of engagement. Esteban-Millat et al. (2018) identified flow a reliable factor for exploring learners' intrinsic motivation in e-learning. When students find pleasure in e-learning interactions, they are more inclined to participate actively, thereby positively influences task implementation (Esteban-Millat et al., 2018).

#### Social support

Social support refers to “social interactions or relationships that provide individuals with actual assistance or a feeling of attachment to a person or a group that is perceived as caring or loving” (Hobfoll and Stokes, 1988, p. 467). There are two common forms of classification for social support. One pertains to providing assistance (e.g., instrumental, emotional, and experiential) (Lin and Kishore, 2021; Ruzek et al., 2016). The other classification revolves around the social identity of the supporter (e.g., teachers, peers, and parents) (Gao et al., 2023; Zimet et al., 1988), which was the approach in our study. In OCEL, students encounter challenges related to e-learning tools, apps, and acquiring new knowledge, making social support inevitable. Teachers can aid students in fulfilling the three basic psychological needs of SDT, significantly impacting their autonomy and competence, while peers seem to have a greater impact on students' relatedness (Vasconcellos et al., 2020). Although social support may not always provide students with direct problem-solving skills, it is vital for achieving students' psychological needs (He et al., 2023). In e-learning environments, online communication tools enables students to obtain social support, helping them break free from the sense of loneliness associated with solitary learning.

### Research model and hypotheses

**Figure 1:**
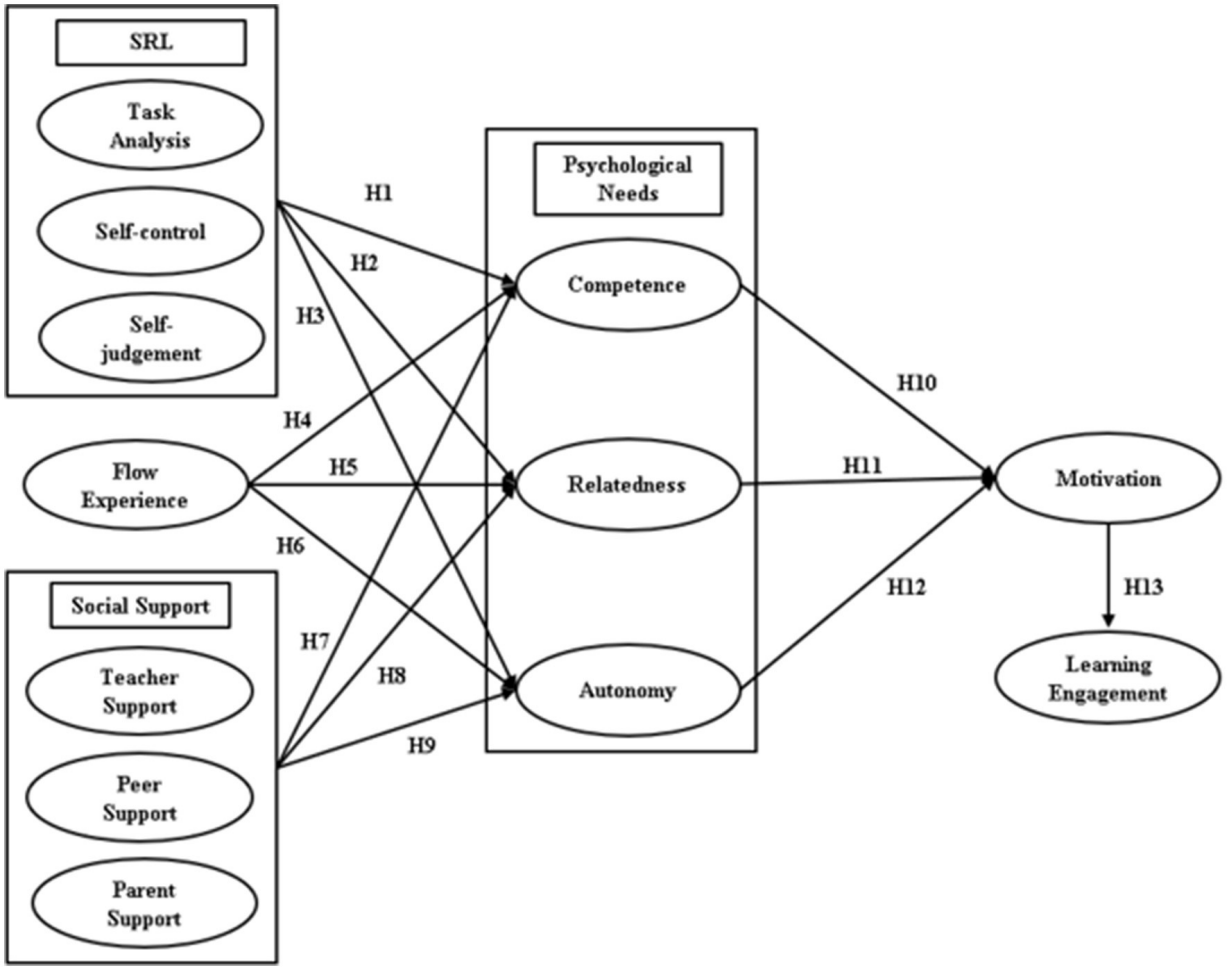
The conceptual model.

Our literature review identified antecedents (SRL, flow experience, and social support) that influence students' basic psychological needs—*competence, relatedness*, and *autonomy*—according to SDT, as well as the resulting consequences (learning engagement). These antecedents mediate student learning engagement in OCEL environments through SDT. Consequently, we proposed a conceptual model that follows a sequence centered on SDT (Figure 1). In this model, the latent variable corresponding to the arrowhead is assumed to predict the latent variable corresponding to the arrowtail.

#### Social support and basic psychological needs

According to SDT, everyone has an inherent desire toward goodness, and social support can promote or hinder this attribute (Jeno et al., 2019). Positive learning engagement occurs when students' basic psychological needs are supported (Jeno et al., 2019). In empirical research on the application of SDT in physical education, Vasconcellos et al. (2020) found a close relationship between teachers and students' autonomy and competence, while peers determined the strength of students' relatedness. Teachers' autonomy support enables better control over learning direction and actions among college students (Flanigan et al., 2023). Roca and Gagné (2008) and Luo et al. (2021) considered individuals' need for relatedness as their need to feel the connection and support of others (e.g., parents, teachers, and peers). SDT emphasizes the positive impact of receiving support from one's environment in meeting individuals' psychological needs (Zainuddin, 2018). Ruzek et al. (2016) have further confirmed that teachers initially providing emotional support to students can lead to enhanced control competence in later learning, fostering students' willingness to exercise initiative in daily activities and establish strong interpersonal relationships. During adolescence, peer interaction play a critical factor promoting the growth of individuals by developing autonomy through peers' communication (Csikszentmihalyi, 2014). Grusec and Goodnow (1994) proposed that, for individuals to internalize, they must accept the clear information and support provided by their communities to promote their autonomy. The following hypotheses were consequently formulated:

*H*_1_: Student competence is positively influenced by social support.

*H*_2_: Student relatedness is positively influenced by social support.

*H*_3_: Student autonomy is positively influenced by social support.

#### Flow experience and basic psychological needs

Flow experience, a concept rooted in positive psychology, arises when individuals engage in specific tasks, contributing to the improvement of individuals' competence in knowledge (Rosas et al., 2023)—a notion that aligns with our research context. Individuals immersed in a state of flow have a keen awareness of their performance, and those with lower skills can improve their competence to perform a certain activity through constantly being in the flow state (Csikszentmihalyi, 2014). Flow experience is related to individual autonomy–individuals with high autonomy tend to maintain a positive psychological state when performing specific activities (Hong et al., 2019). The greater tasks set by individuals, the longer time invested in them, underscoring how flow experience can protect and guide individuals' autonomy (Czikszentmihalyi, 1990). Flow experience may also be influenced by external evaluations, with positive statements serving as catalysts for promoting an individual's flow experience (Czikszentmihalyi, 1990; Luo et al., 2021). This enables individuals to maintain a clear mind, steering them away from self-indulgence. This study set the following hypotheses:

*H*_4_: Student competence lies in the influence of student flow experience.

*H*_5_: Student relatedness lies in the influence of student flow experience.

*H*_6_: Student autonomy lies in the influence of student flow experience.

#### SRL and basic psychological needs

SRL stands as an important skill that facilitates students' self-direction. In e-learning, SRL plays a pivotal role in students achieving goals and improving their learning autonomy through the continual adjustment of learning strategies in practice (Gabriel et al., 2020; Papamitsiou and Economides, 2019). Papamitsiou and Economides (2019) demonstrated through empirical evidence that the continuous practice of SRL has a positive impact on the development of autonomy. Relatedness involves collaboration among students and familiar individuals in SRL **(**Zainuddin, 2018). Learners in e-learning must connect with others when facing complex topics, solve challenging problems that they are not adept at in SRL, and receive assistance from peers (Wong et al., 2018). Luo et al. (2021) indicated that SRL skills reflect students' basic psychological needs for competence, thereby facilitating the ability of individuals aspiring to be proficient in ICT activities to effectively utilize online tools to achieve goals. Furthermore, Zheng et al. (2018) found that SRL may enhance students' competence in mobile learning. This study set the following hypotheses:

*H*_7_: Student relatedness is positively linked to student SRL.

*H*_8_: Student autonomy is positively linked to student SRL.

*H*_9_: Student competence is positively linked to student SRL.

#### Motivation and basic psychological needs

The core element of SDT lies in motivation, serving as the primary impetus for action (Sørebø et al., 2009). Individuals dream of success and approach their goals without hesitation. However, when the expected performance cannot be achieved, individuals' motivation weaken until *a motivation* occurs (Englund et al., 2023). Students exhibit greater autonomy and intrinsic motivation when engaging in activities of their choice (Dutt et al., 2023; Englund et al., 2023). In mathematics education, students driven by autonomous motivation demonstrate more positive learning engagement and outcomes, where autonomy and relatedness in basic psychological needs exhibit a weaker impact on their motivation compared to competence (Wang et al., 2022). Students who receive support for learning strategies can be provided with opportunities for autonomy, potentially reshaping their motivational tendencies (Chiu, 2021). Luo et al. (2021) conducted empirical research on students' learning motivation in an online SRL environment, and found that their relatedness and competence impact extrinsic motivation, while the three basic needs are related to intrinsic motivation. This study formulated the following hypotheses:

*H*_10_: Student competence will predict student motivation.

*H*_11_: Student relatedness will predict student motivation.

*H*_12_: Student autonomy will predict student motivation.

#### Motivation and learning engagement

In this study, learning engagement was characterized as a dynamic and collaborative system constructed by students based on their personal goals. SDT assumes that the learning environment either supports or thwarts students' autonomy, competence, and relatedness, thereby stimulating their intrinsic and extrinsic motivation. Conversely, student motivation determines their approach to learning (Englund et al., 2023). Sun and Rueda (2012) found that students driven by motivational factors tend to exhibit high levels of learning engagement in online education. Whether in traditional or digital learning approaches, learners with stronger self-directed learning competence tend to have stronger learning motivation (Chai et al., 2023). Students equipped with sufficient motivational factors must also possess competence to achieve motivation–when combined, these elements enable students to engage successfully in learning (Lai et al., 2021). When students' intrinsic or extrinsic motivations align with competence, their learning engagement will increase (Csikszentmihalyi, 2014). Individuals' intrinsic emotional motivation can influence their comprehension and action capabilities (Tripon, 2023). Moon and Ke (2019) found that in a gamified learning environment, student engagement often varies based on their previous motivation. Stimulating student engagement depends on assisting students in developing different motivations; a sound motivation mechanism predicts higher student engagement (Chiu, 2021). This study set forth the following hypothesis:

*H*_13_: Student motivation effects student learning engagement.

## Methods

### Participants

Participants in our study were required to possess an OCEL context, with eligibility assessed based on age range (18–22 years), good health status, relevant academic background, and signed informed consent. We carefully screened students to identify those who were suitable potential students for our research context. To target this student group, we organized extensive gatherings, both online and offline, engaging over 3,000 students from WeChat^®^ groups, QQ^®^ groups, and offline classrooms across five Chinese universities, From this pool, we selected 711 students for further participation. Following a pre-testing with 50 students, the questionnaire items were evaluated based on feedback regarding their clarity, relevance, and completeness. The evaluation criteria included student understanding, response consistency, and the identification of any ambiguous or confusing questions. Consequently, several questions were either eliminated or reworded for enhanced clarity. The review was conducted by a panel of experts in the field, who employed a combination of qualitative analysis and expert consensus to assess and refine the questionnaire. Ultimately, a data cleaning process was conducted to eliminate invalid responses with identical IP addresses or unusually short response times. This resulted in the retention of 593 valid responses, accounting for 82.00% of the total. Table 1 presents the students' demographic information of the students and the time spent on OCEL activities.

**Table 1:** The information of 593 students.

	**Items**	**Frequency**	**Proportion (%)**
Gender	Male	304	51.30
	Female	289	48.70
Major	Data engineering	90	15.20
	Humanities	96	16.20
	Accountancy	109	18.40
	Economics	102	17.20
	Arts	88	14.80
	Management	108	18.20
Time spent in OCEL daily	< 1 h	109	18.40
	1–3 h	251	42.30
	3–5 h	156	26.30
	>5 h	77	13.00

### Instrument

The research instruments utilized in this study included a meticulously constructed questionnaire aimed at gathering data concerning the eight latent variables. The questionnaire was carefully crafted to accurately measure the theoretical dimensions of interest, adhering to the principles of construct validity and reliability. The design process involved a thorough review of current literature, consultations with experts, and pilot testing to refine the questionnaire items, ensuring their clarity and relevance. The latent variables in the proposed model were adapted from well-established scales known for their reliability.

#### SDT scale

The Basic Psychological Need Satisfaction Scale tests overall need satisfaction (Van den Broeck et al., 2011), with *autonomy, competence*, and *relatedness* at the core of its research structure. Autonomy is composed of three items (e.g., “I feel capable of manipulating OCEL according to establish goals”); competence consists of three items (e.g., “I feel capable of OCEL”); and relatedness includes three items (e.g., “I feel close to people related to OCEL”). SDT categorizes motivation into three categories rather than simply intrinsic and extrinsic motivation: *amotivation, intrinsic motivation*, and *extrinsic motivation* (Vasconcellos et al., 2020). Amotivation characterizes individuals who lack drive. In our study, students engaged in OCEL with a certain goal; therefore, there was no amotivation stage. They started OCEL with intrinsic motivation (e.g., “In OCEL, I feel fulfilled and satisfied”) or extrinsic motivation (e.g., “OCEL makes me feel closer to my goal”).

#### SRL scale

The Online Self-Regulated Learning Questionnaire, developed by Barnard et al. (2009), measures students' beliefs about e-learning through six subscales encompassing a total of 24 items. In our research context, the proposed model incorporates goal setting (e.g., “I set goals for different stages in OCEL”), task strategies (e.g. “I develop different strategies for implementing OCEL”), and self-evaluation (e.g., “I evaluate the effectiveness of OCEL from different aspects”) as measurement subscales to align with our research objectives.

#### Flow experience scale

Flow is widely used in various domains such as business and education to assess user experiences concerning the design of products and learning systems. Rosas et al. (2023) found that no consensus exists on the evaluation scale for flow within existing questionnaires. Hong et al. (2019) indicated that the design of the flow scale was adapted from a statement of flow, with all reactions reflecting positive aspects of the flow experience. In this study, three items were set for evaluating flow (e.g., “I believe that I have a high frequency of pleasure engagement in OCEL”).

#### Social support scale

The Multidimensional Scale Perceived Social Support, originally designed by Zimet et al. (1988), has been adapted into multiple language versions, including Chinese and Russian (Lin et al., 2019). Labrague et al. (2021) confirmed the excellent internal consistency and correlation of this scale. In e-learning, social support mainly comes from parents, teachers, and peers (Zimet et al., 1988; Gao et al., 2023). Therefore, in our study, social support comprised three observed variables (e.g., “I feel that parents, teachers, and peers provide me with timely assistance in OCEL”).

#### Learning engagement scale

The model of student engagement developed by Fredricks et al. (2004) and Fredricks et al. (2005) considers that student learning consists of three dimensions– *behavioral, emotional*, and *cognitive*. Sun and Rueda (2012) and Jung and Lee (2018) reported that the Cronbach's α coefficients of the student engagement model were acceptable and satisfactory, respectively. Lam et al. (2016) demonstrated that the model exhibits satisfactory construct validity in various contexts. In our current study, the learning engagement scale consisted of three observed variables (e.g., “I am able to pay attention consistently when I am taking OCEL”).

### Data analysis

A structural equation model (SEM), a statistical method used to analyse causal relationships among variables within a model—was applied to analyse the questionnaire data. SEM serves as a form of regression analysis that allows for the simultaneous examination of multiple predictors and their effects on multiple outcomes, while accounting for measurement errors and latent variables (MacCallum and Austin, 2000). This study used IBM^®^ SPSS^®^ AMOS 26 for SEM to analyse the fit and construct validity of the proposed model, with IBM^®^ SPSS^®^ Statistics 28 utilized to assess the consistency and reliability of the scale.

Throughout the research journey, we encountered several formidable challenges: crafting the questionnaire demanded numerous iterations and the integration of expert insights to guarantee clarity and thoroughness; gathering data posed challenges, particularly concerning sample size and representativeness; and fine-tuning the model required meticulous specification and multiple rounds of optimization. Despite these substantial hurdles, the research culminated in success, offering invaluable insights into the intricate relationships among latent variables.

#### Preliminary analysis

Table 2 presents the descriptive statistics for the eight factors. The mean of the factors ranged from 5.390 to 5.673, with their standard deviation (*SD*) ranging from 0.811 to 0.962. The correlation coefficients of the pairwise Pearson's test factors are listed in Table 2. The correlation coefficients of the pairwise factors were significant at a 0.01 significance level, with social support and learning engagement having the smallest correlation coefficient (*r* = 0.455, *p* < 0.01), while SRL and learning engagement exhibited the strongest correlation coefficient (*r* = 0.852, *p* < 0.01). Therefore, the eight factors in the proposed model demonstrated significant pairwise correlations and were positively correlated.

**Table 2:** The preliminary analysis results of 593 students.

	**Mean**	**SD**	**S**	**FE**	**SRL**	**C**	**R**	**A**	**LE**
S	5.404	0.930	1.000						
FE	5.500	0.843	0.559^**^	1.000					
SRL	5.390	0.852	0.459^**^	0.703^**^	1.000				
C	5.501	0.866	0.493^**^	0.752^**^	0.740^**^	1.000			
R	5.453	0.962	0.508^**^	0.643^**^	0.718^**^	0.702^**^	1.000		
A	5.446	0.937	0.493^**^	0.728^**^	0.831^**^	0.770^**^	0.733^**^	1.000	
M	5.673	0.811	0.530^**^	0.713^**^	0.672^**^	0.729^**^	0.717^**^	0.685^**^	1.000
LE	5.528	0.894	0.455^**^	0.626^**^	0.852^**^	0.671^**^	0.641^**^	0.743^**^	0.584^**^

S, Social Support; FE, Flow Experience; C, Competence; R, Relatedness; A, Autonomy; M, Motivation; LE, Learning Engagement; ^**^p < 0.01.

#### Confirmatory factor analysis

**Figure 2:**
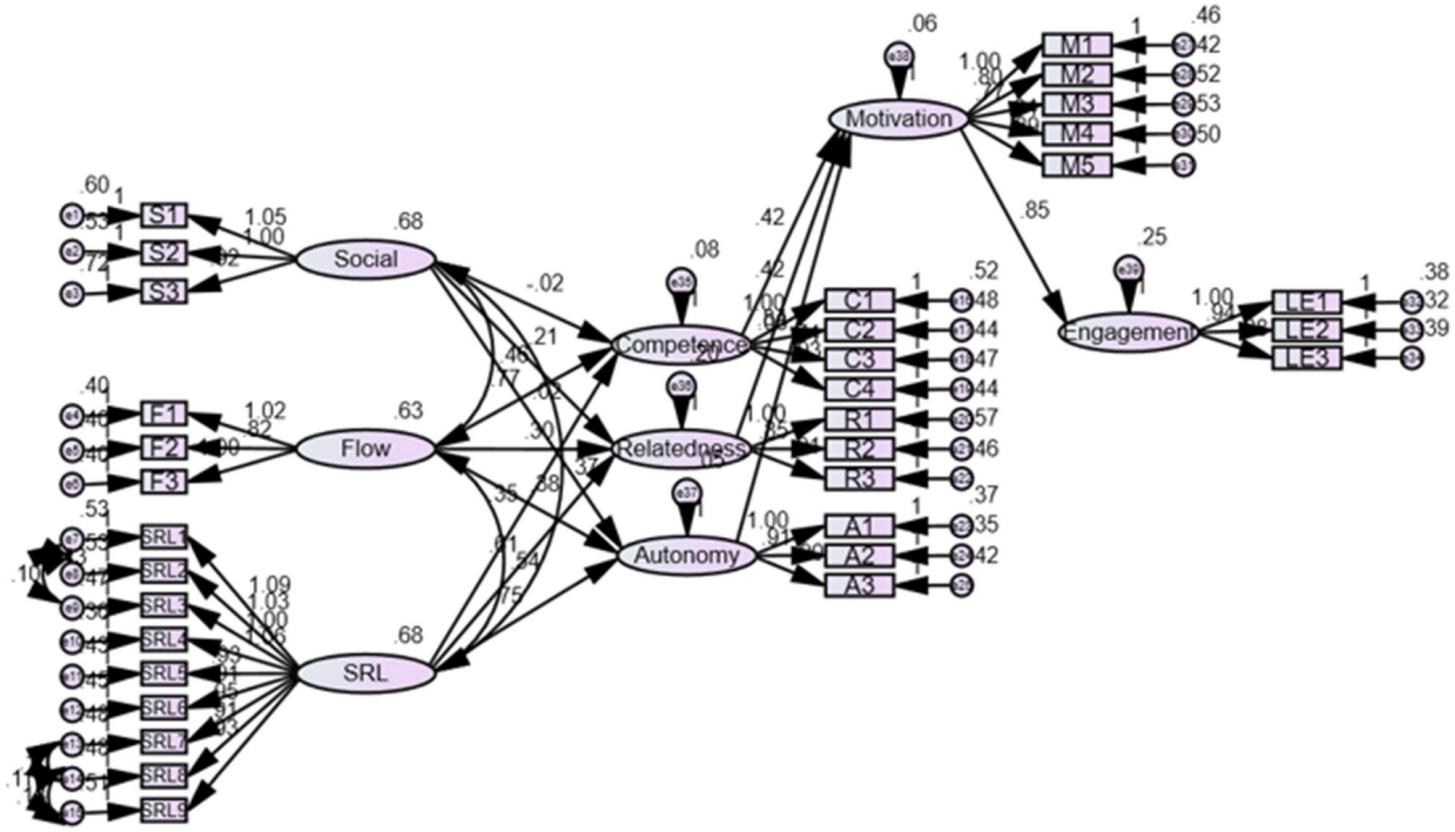
SEM results obtained using AMOS 26.

CFA is used to test and validate the hypothetical factor structure of a measurement instrument, such as a questionnaire (Brown, 2015). CFA determines whether the relationships among observed variables are consistent with the hypothetical factor structure proposed a priori **(**Istiyono, 2019). The analysis involves specifying a measurement model, estimating factor loadings, and evaluating how well the model fits the data. Modifications can be made to the model, if necessary, to improve its goodness of fit. Typically, factor loadings of no < 0.7 are satisfactory (Black et al., 2010). However, Chin et al. (1997) posited that the factor loadings of the adjusted model could be reduced to more than 0.6. To achieve a robust model fit, three items with factor loads below 0.6 in social support, two items in self-regulated learning, and three items on motivation were removed. Table 3 illustrates the factor loadings, with values exceeding 0.6 deemed acceptable. The model fit is shown in Table 4, Line 2, and depicted in Figure 2. χ^2^/*df* (2.888) was below the recommended threshold of 3, which was satisfactory; the root mean square error of approximation (RMSEA; 0.056) and the standardized root mean square residual (SRMR; 0.052) were below the recommended threshold of 0.06. Additionally, Comparative Fit Index (CFI; 0.933), Tucker-Lewis Index (TLI; 0.925) and Incremental Fit Index (IFI; 0.933) surpassed the ideal threshold of 0.9. The Goodness-of-Fit Index (GFI; 0.873) and the Adjusted Goodness-of-Fit Index (AGFI; 0.850) were no < 0.85, indicating acceptability (Hayes, 2013; Kline, 2011; MacCallum et al., 1996). Therefore, the goodness-of-fit indices of the eight-factor model indicated that the model fit is within the recommended ranges (Hayes, 2013), affirming that the proposed model demonstrates a strong fit.

**Table 3:** SEM verification results of the proposed model.

**Latent variables**	**Observed variables**	**Factor loadings**	**SMC**	**CR**	**AVE**	**Cronbach's α**
Social support	S1	0.744	0.444	0.764	0.519	0.760
	S2	0.749	0.554			
	S3	0.666	0.561			
Flow experience	FE1	0.788	0.621	0.808	0.584	0.810
	FE2	0.719	0.517			
	FE3	0.784	0.615			
SRL	SRL1	0.776	0.602	0.926	0.581	0.930
	SRL2	0.759	0.576			
	SRL3	0.769	0.591			
	SRL4	0.826	0.682			
	SRL5	0.762	0.581			
	SRL6	0.747	0.558			
	SRL7	0.748	0.560			
	SRL8	0.732	0.536			
	SRL9	0.734	0.539			
Competence	C1	0.781	0.610	0.838	0.564	0.839
	C2	0.738	0.545			
	C3	0.707	0.500			
	C4	0.776	0.602			
Relatedness	R1	0.819	0.671	0.822	0.607	0.824
	R2	0.730	0.533			
	R3	0.786	0.618			
Autonomy	A1	0.836	0.699	0.856	0.665	0.856
	A2	0.818	0.669			
	A3	0.792	0.627			
Motivation	M1	0.777	0.604	0.842	0.516	0.864
	M2	0.719	0.517			
	M3	0.668	0.446			
	M4	0.695	0.483			
	M5	0.727	0.529			
Learning engagement	LE1	0.815	0.664	0.847	0.648	0.845
	LE2	0.824	0.679			
	LE3	0.776	0.602			

**Table 4:** The model fit of different number of factors.

**Model**	**χ2**	***df***	**χ2/*df***	**RMSEA**	**SRMR**	**GFI**	**AGFI**	**CFI**	**TLI**	**IFI**	**vs**.	***Δχ*2**	**Δ*df***
Threshold values	–	–	< 3	< 0.06	< 0.06	≥0.85	≥0.85	>0.9	>0.9	>0.9	–	–	–
1	1368.682	474	2.888	0.056	0.052	0.873	0.850	0.933	0.925	0.933	–	–	–
2	1710.367	484	3.534	0.065	0.055	0.841	0.816	0.908	0.900	0.908	1 vs. 2	341.685^***^	10
3	1876.469	490	3.830	0.069	0.056	0.819	0.793	0.896	0.888	0.896	2 vs. 3	166.102^***^	6
4	2372.494	493	4.812	0.080	0.056	0.735	0.698	0.859	0.849	0.860	3 vs. 4	496.025^***^	3
5	2456.546	495	4.963	0.082	0.056	0.725	0.689	0.853	0.843	0.853	4 vs. 5	84.052^***^	2

1, Eight-factor model; 2, Seven-factor model; 3, Five-factor model; 4, Three-factor model; 5, Single-factor model; ^***^p < 0.001.

#### Construct validity and construct reliability

Construct validity is important in psychological and educational measurements, ensuring that the tests and measures accurately capture the theoretical dimensions of interest (Campbell-Sills and Stein, 2007). It has two crucial components: convergent validity (CV) and discriminant validity (DV). Establishing both CV and DV ensures that the instrument measures its intended constructs without confounding other related but distinct constructs (Deci and Ryan, 2013). An Average Variance Extracted (AVE) of 0.5 or higher satisfactory CV (Hair, 2009). The AVE values are listed in Table 3. The general method for proving DV is that the square root of a certain AVE is greater than the estimated correlation of other structures in the proposed model **(**Fornell and Larcker, 1981). In this analysis, the square root did not satisfy this standard. Consequently, a competitive model comparison method was adopted, gradually reducing the number of factors to exhibit superior DV in the model proposed in this study (Bagozzi and Phillips, 1982) (see Table 4). Through the chi-square test, the goodness-of-fit of models with fewer factors was significantly lower than that of models with more factors. Accordingly, it is reasonable to confirm that the DV of the proposed eight-factor model was satisfactory.

Construct reliability (CR) was used to evaluate the internal consistency of each structure in the proposed model (Ware et al., 1996), a method distinct from the assessment of CV. Cronbach's α, an important indicator of internal consistency, should ideally be not < 0.7 for the values of CR and Cronbach's α (Bagozzi and Yi, 1989). All CR values ranged from 0.764 to 0.926, while Cronbach's α ranged from 0.760 to 0.930. These results indicated that the construction reliability of the proposed model was ideal.

#### Hypothesis testing

SEM technology tested 13 hypotheses using IBM^®^ SPSS^®^ Amos 26. SEM facilitated the examination of both direct and indirect effects of the latent variables (Hayes, 2013). The results are summarized in Table 5. The significance levels for *H*_1_, *H*_3_, and *H*_12_ were 0.706, 0.714, and 0.424, respectively, indicating that *H*_1_, *H*_3_, and *H*_12_ were rejected. The significance levels for *H*_2_ and *H*_5_ were 0.001 and 0.005, respectively. Therefore, at a 0.01 significance level, *H*_2_ and *H*_5_ were supported. *H*_4_, *H*_6_, *H*_7_, *H*_8_, *H*_9_, *H*_10_, *H*_11_, and *H*_13_ were all significant at the 0.001 level, indicating that all these hypotheses were accepted.

**Table 5:** The path direct effect.

**Hypothesis**	**Path of action**	**std**.	**unstd**.	**S.E**.	***t* value**	***p* value**	**Results**
H1	C←S	0.020	−0.022	0.059	−0.377	0.706	Rejected
H2	R←S	0.180	0.207	0.063	3.261	0.001	Accepted
H3	A←S	0.016	0.018	0.050	0.366	0.714	Rejected
H4	C←FE	0.682	0.773	0.114	6.788	^***^	Accepted
H5	R←FE	0.256	0.304	0.108	2.818	0.005	Accepted
H6	A←FE	0.330	0.382	0.087	4.387	^***^	Accepted
H7	R←SRL	0.535	0.612	0.083	7.393	^***^	Accepted
H8	A←SRL	0.669	0.748	0.069	10.896	^***^	Accepted
H9	C←SRL	0.320	0.350	0.078	4.498	^***^	Accepted
H10	M←C	0.447	0.416	0.094	4.416	^***^	Accepted
H11	M←R	0.473	0.421	0.069	6.103	^***^	Accepted
H12	M←A	0.086	0.078	0.097	0.800	0.424	Rejected
H13	LE←M	0.818	0.849	0.052	16.410	^***^	Accepted

^***^p < 0.001.

We used Hayes' (2009) bootstrap method to examine potential mediating effects between social support and learning engagement, flow experience and learning engagement, as well as SRL and learning engagement. In AMOS, testing for mediation effects, bootstrapping was set at a 95% confidence interval (CI), conducting 5,000 repeated samples for analysis (see Table 6).

**Table 6:** Standardized bootstrap mediation effect test.

**Path**	**Effect**	**SE**	**Bias-corrected 95%CI**		

			**Lower**	**Upper**	***P***
S → C → M → LE	−0.008	0.039	−0.076	0.053	0.707
S → R → M → LE	0.074	0.037	0.011	0.156	0.025
S → A → M → LE	0.001	0.020	−0.011	0.037	0.605
FE → C → M → LE	0.249	0.140	0.056	0.526	0.025
FE → R → M → LE	0.099	0.052	0.018	0.223	0.017
FE → A → M → LE	0.023	0.098	−0.054	0.360	0.446
SRL → C → M → LE	0.117	0.074	0.010	0.308	0.028
SRL → R → M → LE	0.207	0.063	0.102	0.364	0.001
SRL → A → M → LE	0.047	0.109	−0.118	0.369	0.500

Bootstrap CI excluded 0, indicating that the indirect effect was significant. The indirect effect of S → C → M → LE was −0.008, *SE* was 0.039, *p* value was 0.707, and the bootstrap 95% CI generated by this path contained 0, indicating that the mediating effect between social support and learning participation through C and M was not significant. The explanation of the three paths S → A → M → LE, FE → A → M → LE and SRL → A → M → LE is similar to the S → C → M → LE path. The indirect effect of S → R → M → LE was 0.074, *SE* was 0.037, and *p* value was 0.025. The bootstrap 95% CI generated by this pathway excluded 0, indicating that both the direct and total effects of social support on learning engagement through R and M were significant. The four paths FE → C → M → LE, FE → R → M → LE, SRL → C → M → LE and SRL → R → M → LE are explained similarly to the S → R → M → LE path.

## Results and discussion

The purpose of this study was to explore the impact of SRL, flow experience, and social support on the main SDT framework in the OCEL context, as well as the impact of student motivation on learning engagement. Particularly, we examined the mediating role of SDT in student learning engagement. Out of the 13 hypotheses proposed for the direct path test, 10 hypotheses were supported and three hypotheses were rejected. In the indirect path test, five hypotheses yielded significant results, while four hypotheses did not demonstrate significance.

The first three proposed hypotheses (*H*_1_, *H*_2_, and *H*_3_), social support was found to significantly influence the relatedness aspect of SDT, aligning with its impact on students in ICEL as indicated by Roca and Gagné (2008), Zainuddin (2018), and Luo et al. (2021). However, the impact of social support on the competence aspect of SDT was not significant, contrasting with the findings of Wang et al. (2022). This may be attributed to differences between OCEL and ICEL, where students in OCEL often engage in self-directed learning, leading to competence being an intrinsic motivation that is less related to social support. Additionally, competence and self-efficacy are distinct constructs (Deci and Ryan, 2013). Moreover, the impact of social support on the autonomy aspect of SDT was also insignificant, consistent with the findings drawn by Van den Broeck et al. (2011), Zainuddin (2018), and Papamitsiou and Economides (2019) in the ICEL context. Van den Broeck et al. (2011) highlighted the distinction between autonomy in SDT and in organizational psychology, emphasizing that the former embodies an individual's subjective sense of freedom of choice, which may be compromised when individuals conform to external demands. Similarly, Papamitsiou and Economides (2019) posited that autonomy represents an individual's volition, which can often be constrained by external factors.

Our results provided support for *H*_4_, *H*_5_, and *H*_6_, indicating that students' flow experience can directly predict the three psychological needs of SDT, consistenting with the ICEL context. In the analysis of the mediating effect of SDT, student flow experience mediated student learning engagement through SDT. However, the mediating effect of SDT was only significant at 0.05 significance level suggesting a limited mediating effect and emphasizing the importance of direct effects in the analysis. In the OCEL context, students' flow experience enhanced their basic psychological needs—*competence, relatedness*, and *autonomy*—in SDT, owing to the interconnectedness between flow experience and SDT in evoking psychological responses. Csikszentmihalyi (2014) posited that flow experience is closely related to psychological factors. Additionally, Rosas et al. (2023) proposed that individuals, while engaged in specific tasks, undergo a pleasurable psychological state characterized by experiencing personal growth. Consequently, the successful fulfillment of the three basic psychological needs outlined in SDT was achieved.

Wong et al. (2018) revealed that SRL is associated with distinct learner traits in various learning contexts. In our study, we confirmed and supported SRL's influence on *competence, relatedness*, and *autonomy* within the framework of SDT. Building upon the findings of Papamitsiou and Economides (2019) and Liao et al. (2023) from prior studies in the ICEL setting, it was demonstrated that SRL positively impacts autonomy, with validation provided through four SRL strategies. Papamitsiou and Economides (2019) highlighted the inherent relationship between SRL and autonomy, considering them essentially overlapping concepts, thereby shedding light on why SRL exerts a positive influence on autonomy. Zainuddin (2018) indicated that autonomy is highly correlated with SRL, suggesting that engaging students in OCEL activities where they primarily master content independently can help boost their critical thinking competence. Zheng et al. (2018) agreed that SRL is beneficial for improving student competence. We found that the relationship between SRL and relatedness is rarely found in existing literature, and the connection is only found in descriptions.

According to SDT, motivation can only be achieved when all three basic psychological needs are met (Van den Broeck et al., 2016), but this does not appear to be the case. Luo et al. (2021) found that the three needs of SDT have varying impacts on motivation because of different research contexts. In the workplace, relatedness is not related to intrinsic motivation, while extrinsic motivation is influenced by autonomy. The relatedness of the teachers' context is not related to any motivation, and their autonomy is also unrelated to extrinsic motivation. In our research findings, autonomy was not significant for motivation. Furthermore, all mediating effects along this path were also not significant; neither flow experience nor SRL affected student learning engagement through autonomy and motivation. For instance, the lack of a significant relationship between autonomy and motivation aligns with the findings of Luo et al. (2021), contradicting those of Zainuddin (2018). Zhou (2016) shared a similar perspective. As a result, hypotheses *H*_10_ and *H*_11_ were validated in our study.

The influence of student motivation on student learning engagement has been extensively studied and acknowledged in existing literature (Chai et al., 2023; Gabriel et al., 2020; Luo et al., 2021; Ryan and Deci, 2020; Sun and Rueda, 2012), a correlation that our study also corroborated. Zainuddin (2018) examined the impact of intrinsic and extrinsic motivation on student learning engagement, finding that motivation not only enhanced engagement but also improved students' academic performance. Liao et al. (2023) proposed that learning engagement is a dynamic mechanism involving both personal and contextual factors, with motivation being a personal factor that affects student learning engagement. Sun and Rueda (2012) validated the impact of students' learning motivation on their engagement in e-learning (e.g., behavioral, emotional, and cognitive).

## Conclusion and implications

This study considered students' purposeful self-directed OCEL as the research context, using SDT as the theoretical framework to introduce SRL, flow experience, and social support. The goal was to uncover factors that directly or indirectly influence students' learning engagement within this specific learning environment. This research contribution lies in distinguishing ICEL and OCEL, rather than conflating them. We examined the impact of SRL, flow experience, and social support on students in the context of OCEL, while also comparing our findings with existing research to pinpoint any notable discrepancies.

Our results showed that social support did not significantly impact SDT competence and SDT autonomy, and SDT autonomy did not significantly impact SDT motivation. We also explored the mediating role of SDT in the proposed model and found that the pathways with no significant impact mentioned above were not significant in the indirect pathways of S → C → M → LE, S → A → M → LE, FE → A → M → LE, SRL → A → M → LE. The other mediating effects in this model were only significant at 0.05, indicating that direct effects in this model had a greater impact than indirect effects. Thus, improving students' learning engagement in OCEL should focus more on direct influencing factors.

According to SDT, SRL, social support, flow experience, and our research results, we suggest the following implications for students. First, students should clarify their learning objectives and stimulate intrinsic and extrinsic motivation, by concretising goals, such as improving English reading comprehension within 3 months. Second, before learning, self-assessments can help students identify their weaknesses and track learning progress. By understanding their learning preferences and styles through tests or habit evaluations, students can tailor their study plans accordingly. Subsequently, based on the self-assessment results, students should develop a specific learning plan, including scheduling, content selection, and methods. For example, a student can develop a plan to study for 2 h a day, choose online courses or resources as learning content, and use methods such as watching videos, answering exercise questions, and participating in discussions to learn. Seeking social support during the learning process can provide students assistance and facilitate maintenance of their motivation. Engaging in online learning communities, exchanging experiences with other peers, sharing learning resources, and organizing online learning groups for mutual support and encouragement are beneficial strategies. To achieve a state of flow, students should select learning tasks that align with their interests and abilities. By tackling challenging assignments, actively problem-solving, and exploring new areas of study, students can immerse themselves in a fulfilling learning experience. A suitable learning environment is crucial for a flow experience. Students should promptly identify and correct their mistakes. Reflecting on their learning processes and results enables students to identify ways for improvement. Lastly, maintaining patience, perseverance, and a commitment to continuous improvement are essential for enhancing learning effectiveness over time. By implementing these strategies, students can optimize their learning journeys and achieve greater success in their educational pursuits.

## Limitations and future research

This study is subject to several limitations that warrant consideration. First, the questionnaire data was collected from five universities in China, indicating a regional specificity. To enhance the generalizability of findings, future research could expand data collection nationwide, encompassing diverse student populations. Second, our research was conducted in the OCEL context. Comparisons with ICEL context studies may introduce common method bias due to scale differences. Future work could collect data from both contexts using consistency scales to form control group and enable more objective analysis. Third, individual differences among students, such as learning and cognitive competence, can significantly impact students' self-directed learning in OCEL. Although students were screened, these differences remain unknown. Future study could validate individual abilities and address these differences. Lastly, students set ambitious and simple goals differently. The former tests more psychological factors than the latter, leading to significant fluctuations in the flow experience and basic psychological needs, as simple goals result in inapparent experiences. Future research could target comparable students to minimize such disparities and ensure more consistent analysis.

## Data Availability

The raw data supporting the conclusions of this article will be made available by the authors, without undue reservation.
